# Focal adhesion kinase-dependent focal adhesion recruitment of SH2 domains directs SRC into focal adhesions to regulate cell adhesion and migration

**DOI:** 10.1038/srep18476

**Published:** 2015-12-18

**Authors:** Jui-Chung Wu, Yu-Chen Chen, Chih-Ting Kuo, Helen Wenshin Yu, Yin-Quan Chen, Arthur Chiou, Jean-Cheng Kuo

**Affiliations:** 1 Institute of Biochemistry and Molecular Biology, National Yang-Ming University, Taipei 11221, Taiwan; 2 Institute of Biophotonics, National Yang-Ming University, Taipei 11221, Taiwan; 3 Biophotonics and Molecular Imaging Research Center, National Yang-Ming University, Taipei 11221, Taiwan

## Abstract

Directed cell migration requires dynamical control of the protein complex within focal adhesions (FAs) and this control is regulated by signaling events involving tyrosine phosphorylation. We screened the SH2 domains present in tyrosine-specific kinases and phosphatases found within FAs, including SRC, SHP1 and SHP2, and examined whether these enzymes transiently target FAs via their SH2 domains. We found that the SRC_SH2 domain and the SHP2_N-SH2 domain are associated with FAs, but only the SRC_SH2 domain is able to be regulated by focal adhesion kinase (FAK). The FAK-dependent association of the SRC_SH2 domain is necessary and sufficient for SRC FA targeting. When the targeting of SRC into FAs is inhibited, there is significant suppression of SRC-mediated phosphorylation of paxillin and FAK; this results in an inhibition of FA formation and maturation and a reduction in cell migration. This study reveals an association between FAs and the SRC_SH2 domain as well as between FAs and the SHP2_N-SH2 domains. This supports the hypothesis that the FAK-regulated SRC_SH2 domain plays an important role in directing SRC into FAs and that this SRC-mediated FA signaling drives cell migration.

Cell migration involves the extension of a cell protrusion; this is followed by the attachment of the protrusion to the substratum at the cell front, the translocation of the cell body and, finally, the detachment of the trailing end of the cell from the substratum^1^,^2^,^3^. During this process, integrin-based adhesive organelles, focal adhesions (FAs), need to dynamically control the coupling of actin filaments and the extracellular matrix (ECM) in order to translate actin polymerization and actomyosin contraction into cell motion^4^,^5^,^6^. FAs are complexes that contain hundreds of proteins; these proteins include structural, signaling and scaffold proteins that are able to link the actin cytoskeleton to clustered transmembrane integrin receptors^7^. It has been well established that there is a hierarchical cascade that involves FA compositional changes during FA maturation^8^. During this process, the FAs grow in size and undergo the spatiotemporal transduction of distinct biological signals. The latter occurs via specific groups of proteins found within the FAs that are able to control actin cytoskeleton organization and thus drive cell migration^8^,^9^,^10^.

One of the mechanisms regulating the dynamic organization of FAs involves the activity of tyrosine-specific kinases and phosphatases within the FAs. These tyrosine-specific kinases and phosphatases regulate the phosphorylation status of their substrates; such phosphorylation creates docking sites for Src-homologue-2 (SH2) domains [phosphotyrosine (pY) binding domains] on appropriate interacting molecules^11^. Thus, within FAs, the transient localization of tyrosine-specific kinases and phosphatases is likely to play a key role in dynamically controlling the association of other SH2 domain-containing FA proteins within FAs; this will regulate the composition of these FAs and control FA-mediated signaling. Among the tyrosine-specific kinases and phosphatases within FAs, the following proteins, SRC, SHP1 and SHP2, have been found to contain SH2 domain^7^. SRC, a non-receptor protein tyrosine kinase, contains an N-myristoylation site as well as SH3, SH2 and kinase domains (Fig. 1a)^12^,^13^. The SH2 domain of SRC interacts with its C-terminal phospho-tyrosine (Y527) to form a closed, catalytically inactive, conformation^12^,^14^,^15^,^16^. This can be opened and activated by dephosphorylating of Y527 (or Y530 in human SRC)^17^,^18^,^19^ or by the binding of the SH2 or SH3 domain to another protein^20^,^21^,^22^,^23^,^24^,^25^,^26^,^27^. Active SRC is able to initiate intercellular signaling via SH2-dependent or SH3-dependent binding to its downstream substrates, which include FAK or p130Cas. This initiates a process of phosphorylation and activation that results in integrin-mediated adhesion signaling and cell motility^28^,^29^,^30^. The SHP1 and SHP2 phosphatases, which are non-receptor protein tyrosine phosphatases, contain two SH2 domains (called N-SH2 and C-SH2) and a phosphatase domain (PTPase) (Fig. 1a)^31^,^32^,^33^; these are known to dephosphorylate downstream substrates that are able to modulate FAs. For example, a reduction in α-actinin phosphorylation by SHP1^34^ or SHP2^35^,^36^ promotes its binding to actin and thereby triggers the association of α-actinin with FAs; this strengthens the links between integrins and the actin cytoskeleton. SHP2 also down-regulates the tyrosine phosphorylation of ROCKII at Y722 resulting in the activation of ROCKII and the promotion of FA maturation^37^. From the above studies it is clear that SRC, SHP1 and SHP2 activity within FAs is strongly correlated with the organization of FAs.

Given the importance of SRC, SHP1 and SHP2 to FA organization, the mechanism directing these enzymes transiently to FAs, whether the SH2 domain is able to control the targeting of the enzymes to FAs and the association of these enzymes with various substrate proteins remains unclear. Previous studies have implied that the SH2 domain is important to the directing of SRC to FAs because SRC mutants with an accessible SH2 domain are localizable at FAs^12^,^38^. Indeed, SRC kinase-domain deletion mutants with a point mutation within the SH2 domain (R175L) are unable to associate with FAs in SYF (SRC^−/−^, Yes^−/−^, Fyn^−/−^) MEFs^39^, indicating that the association between SRC and FAs in SYF cells depends on the protein having a functional SH2 domain. However, in cells that express SRC-family kinases, this SRC mutant (R175L) has been reported to be localized with FAs^12^, revealing that the role that the SH2 domain of SRC plays in FA targeting remains controversial. Although SHP1 and SHP2 contain SH2 domains, it is not clear whether these SH2 domains are able to mediate the association of these phosphatases with FAs. Given the critical role of SRC, SHP1 and SHP2 in integrin-mediated adhesion signaling, we set to investigate whether the SH2 domain is able to direct these tyrosine-specific kinase and phosphatases towards FAs.

In the present study we have examined the localization of SH2 domain constructs derived from SRC, SHP1 and SHP2. We found that the SH2 domain of SRC and N-SH2 domain of SHP2 are able to bring about association with FAs; nevertheless, only the SH2 domain of SRC is able to be regulated by FAK, which enhances the abundance of SRC in FAs. We have also shown that the SH2 domain is necessary and sufficient for SRC recruitment to FAs. We have further demonstrated that the association of SRC with FAs plays a key role in transducing SRC-mediated signals, in the promotion of FA formation/maturation and in enhancing cell migration.

## Results

### The SRC_SH2 domain and SHP2_N-SH2 domain are able to associate with focal adhesions

**Figure 1:**
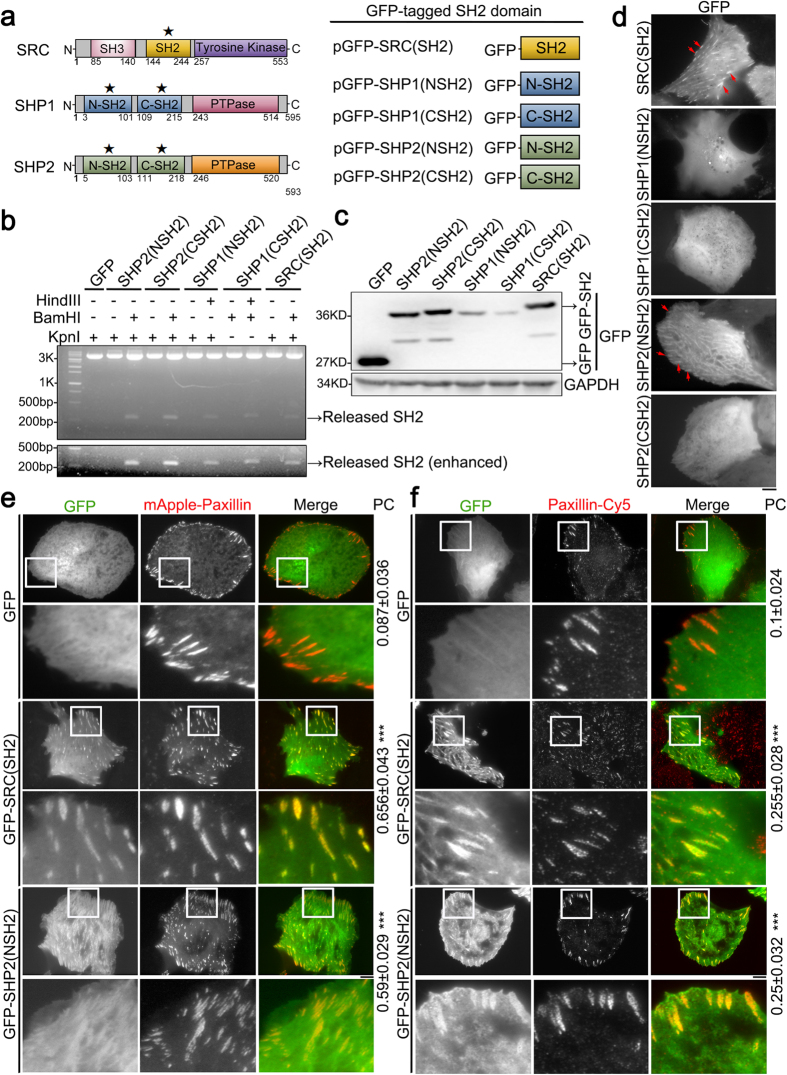
The SRC_SH2 and SHP2_N-SH2 domains associate with focal adhesions. (**a**) Diagram of the domain structures of SRC, SHP1 and SHP2. The SH2 domains of SRC, SHP1 and SHP2 were cloned into pGFP-C1 individually and the resulting plasmids were named pGFP-SRC(SH2), pGFP-SHP1(NSH2), pGFP-SHP1(CSH2), pGFP-SHP2(NSH2) and pGFP-SHP2(CSH2). (**b**) DNA from pGFP-C1, pGFP-SHP1(NSH2), pGFP-SHP1(CSH2), pGFP-SHP2(NSH2), pGFP-SHP2(CSH2) and pGFP-SRC(SH2) were digested with the restriction enzymes as indicated, which either linearize the DNA construct or release the DNA insert encoding the SH2 domain from the indicated constructs, as shown on an agarose gel stained with EtBr. Below: the same gel with a longer exposure time. (**c**) U2OS cells transfected with pGFP-C1, pGFP-SHP1(NSH2), pGFP-SHP1(CSH2), pGFP-SHP2(NSH2), pGFP-SHP2(CSH2) or pGFP-SRC(SH2) were analyzed by Western blotting with GFP and GAPDH antibodies. (**d**) TIRFM images of U2OS cells expressing GFP-SRC(SH2), GFP-SHP1(NSH2), GFP-SHP1(CSH2), GFP-SHP2(NSH2) or GFP-SHP2(CSH2). The adhesion-like pattern marked with red arrows in the images. Scale bar, 10 μm. (**e**) TIRFM images of U2OS cells co-transfected with mApple-paxillin (red) and pGFP-C1, pGFP-SRC(SH2) or pGFP-SHP2(NSH2) (green). Bar, 10 μm. The 20 μm × 20 μm areas indicated in the upper images are magnified in the images below. Values indicate Pearson’s correlation coefficients (PC) of images of mApple-paxillin and GFP, GFP-SRC(SH2) or GFP-SHP2(NSH2) in U2OS cells. Data are means ± s.e.m. (GFP: n = 6 cells; GFP-SRC(SH2): n = 6 cells; GFP-SHP2(NSH2): n = 7 cells). ***p < 0.001, in comparison with GFP-expressing cells. (**f**) TIRFM images of U2OS cells transfected with pGFP-C1, pGFP-SRC(SH2) or pGFP-SHP2(NSH2) (green) and immunostained to detect paxillin (red). Bar, 10 μm. The 20 μm × 20 μm areas indicated in the upper images are magnified in the images below. Values indicate PC of images of paxillin-Cy5 and GFP, GFP-SRC(SH2) or GFP-SHP2(NSH2) in U2OS cells. Data are means ± s.e.m. (GFP: n = 11 cells; GFP-SRC(SH2): n = 9 cells; GFP-SHP2(NSH2): n = 9 cells). ***p < 0.001, in comparison with GFP-expressing cells.

Before demonstrating whether SRC, SHP1 and SHP2 are able to localize to FAs via their SH2 domains, we first identified the SH2 domains of these three enzymes, all of which are known to localize at FAs^7^. Previous studies have shown that SRC contains only one SH2 domain, while SHP1 and SHP2 both contain two SH2 domains, one called N-SH2 and the other called C-SH2, as indicated in Fig. 1a. The cDNA of each individual SH2 domain was amplified (Supplemental Table 1) and fused via their C terminus to GFP in order to generate recombinant pGFP-SH2 constructs, namely pGFP-SRC(SH2), pGFP-SHP1(NSH2), pGFP-SHP1(CSH2), pGFP-SHP2(NSH2) and pGFP-SHP2(CSH2) (Fig. 1a,b). The modified GFPs containing these SH2 domains allow the SH2 protein to be expressed as a fluorescent protein that is detectable and visualizable in cells (Fig. 1c,d).

Next we examined the intracellular localization of these various GFP-SH2 proteins by expressing pGFP-SRC(SH2), pGFP-SHP1(NSH2), pGFP-SHP1(CSH2), pGFP-SHP2(NSH2) or pGFP-SHP2(CSH2) individually in human osteosarcoma U2OS cells, which were then plated on fibronectin-coated coverslips. On visualizing the cells by total internal reflection fluorescence microscopy (TIRFM), it was found that GFP-SRC(SH2) and GFP-SHP2(NSH2) did show an adhesion-like pattern (marked with the red arrows), while pGFP-SHP1(NSH2), pGFP-SHP1(CSH2), and pGFP-SHP2(CSH2) were found diffused throughout the cytosol (Fig. 1d). To further examine if SRC(SH2) and SHP2(NSH2) specifically localize within FAs, we utilized TIRFM to image U2OS cells co-transfected with mApple-paxillin, which acted as a FA marker, and pGFP-C1, pGFP-SRC(SH2) or pGFP-SHP2(NSH2). A comparison of images of GFP-expressing cells showed that GFP-SRC(SH2) mainly co-localized with paxillin at FAs close to the cell center and periphery, while GFP-SHP2(NSH2) appeared as fibrillary structures within the cell center; this association included paxillin-marked FAs, and localization within lamellipodia (Fig. 1e). In order to prevent false-positive results that might have been caused by the bleed-through, we immunostained paxillin with Cy5-conjugated secondary antibodies in cells expressing pGFP-C1, pGFP-SRC(SH2) or pGFP-SHP2(NSH2) (Fig. 1f). These images produced similar results, as shown in Fig. 1e. Thus it is highly likely that the SH2 domain of SRC and N-SH2 domain of SHP2 are involved in the association of SRC and SHP2 with FAs, respectively.

### The SH2 domain of SRC is involved in the protein’s association with focal adhesions and this association is regulated by focal adhesion kinase

**Figure 2:**
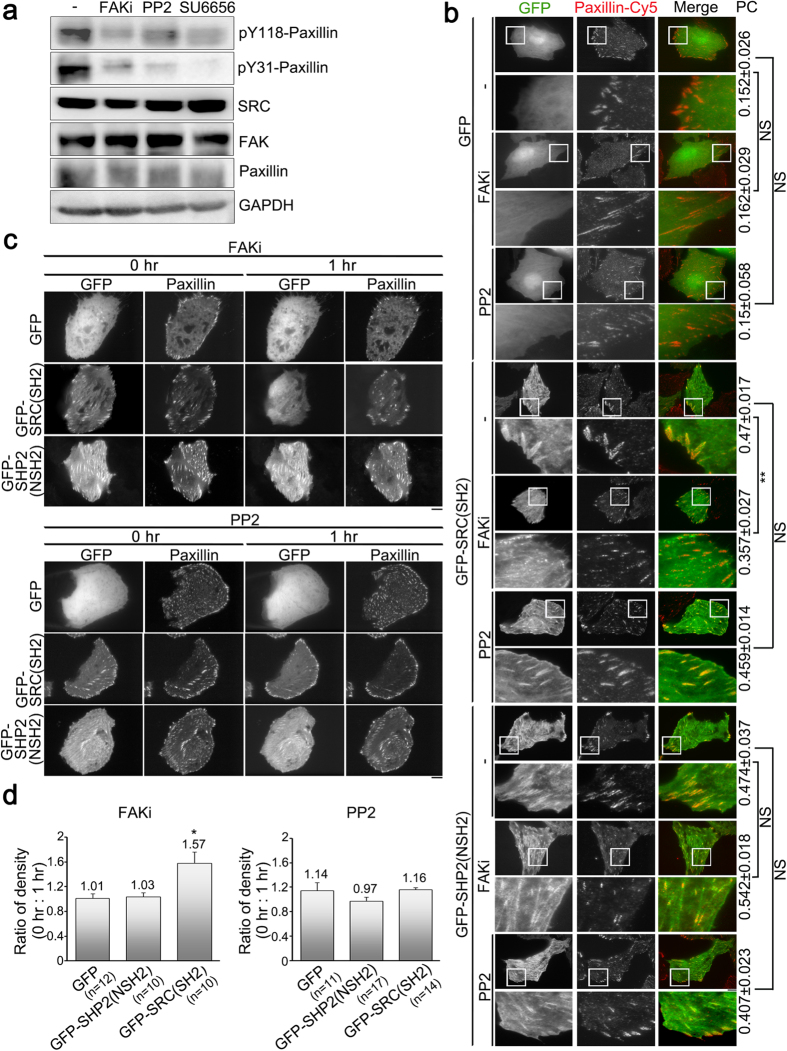
Focal adhesion kinase activity, but not SRC activity, positively regulates the abundance of SRC_SH2 domain within focal adhesions. (**a**) Total cell lysate from untreated U2OS cells (−) or cells treated with FAKi (focal adhesion kinase inhibitor; 50 μM, 1 h), PP2 (SRC inhibitor; 10 μM, 1 h) or SU6656 (SRC inhibitor; 5 μM, 1 h) were analyzed by Western blotting to detect pY118-paxillin, pY31-paxillin, SRC, FAK, paxillin and GAPDH. (**b**) U2OS cells transfected with pGFP-C1, pGFP-SRC(SH2) or pGFP-SHP2(NSH2) were untreated (−) or treated with FAKi or PP2, and immunostained to detect paxillin. TIRFM images of GFP, GFP-SRC(SH2) or GFP-SHP2(NSH2) (green) and paxillin (red). Bar, 10 μm. The 20 μm × 20 μm areas indicated in the upper images are magnified in the images below. Values indicate Pearson’s correlation coefficients (PC) of images of paxillin-Cy5 and GFP, GFP-SRC(SH2) or GFP-SHP2(NSH2) in U2OS cells untreated (−) or treated with FAKi or PP2. Data are means ± s.e.m. (GFP: n = 3 cells (−), n = 4 cells (FAKi), n = 6 cells (PP2); GFP-SRC(SH2): n = 7 cells (−), n = 6 cells (FAKi), n = 7 cells (PP2); GFP-SHP2(NSH2): n = 5 cells (−), n = 5 cells (FAKi), n = 4 cells (PP2)). **p < 0.01; NS, no significance. (**c**) Time-lapse TIRFM images of U2OS cells co-transfected with mApple-paxillin (red) and pGFP-C1, pGFP-SRC(SH2) or pGFP-SHP2(NSH2) (green) during FAKi or PP2 treatment. The time is shown in hour. Bar, 10 μm. (**d**) The ratio of the average density (intensity per μm^2^) of GFP, GFP-SRC(SH2) or GFP-SHP2(NSH2) within segmented paxillin-marked FAs of U2OS cells treated with FAKi or PP2 at 0 h relative to 1 h. Data are means ± s.e.m. (n = number of cells). *p < 0.05.

SRC and FAK, non-receptor protein-tyrosine kinases, are activated by integrin engagement in order to transduce adhesion signaling, which then cooperatively regulates FA dynamics, enabling cell migration^28^,^29^,^40^,^41^,^42^,^43^,^44^. We first used pharmacological manipulation to modify SRC or FAK activity independently in order to determine the involvement of SRC and FAK activity in the association of the SRC_SH2 domain and the SHP2_N-SH2 domain with FAs. By examining the level of pY31-paxillin and pY118-paxillin^45^,^46^ (Fig. 2a), we found that when cells were treated with an FAK inhibitor (FAKi; 50 μM, 1 h), with the SRC-family tyrosine kinase inhibitor PP2 (10 μM, 1 h) or the SRC-specific inhibitor SU6656 (5 μM, 1 h) there was blockage of FAK-mediated and SRC-mediated signaling in U2OS cells, but this change did not affect the expression level of GFP, GFP-SHP2(NSH2), GFP-SHP2(CSH2), GFP-SHP1(NSH2), GFP-SHP1(CSH2) or GFP-SRC(SH2) (Supplemental Fig. 1a,b). Although the blockage of FAK-mediated signaling decreased the total number of FAs, especially large FAs (>2 μm^2^) and medium-sized FAs (0.5 ~ 2 μm^2^) (Supplemental Fig. 1c,d), the purpose of this experiment is not to examine FAK-mediated signaling on FA formation, but rather to determine FAK-mediated signaling on the association of the SH2 domains with FAs. Compared to control cells, when cells expressing pGFP-C1, pGFP-SRC(SH2) or pGFP-SHP2(NSH2) were treated with FAKi in order to inhibit FAK activity significantly, there was blockage of the localization of GFP-SRC(SH2). However, blockage did not occur with either GFP or GFP-SHP2(NSH2), using paxillin-marked FAs (Fig. 2b). Furthermore, the association of GFP, GFP-SRC(SH2) or GFP-SHP2(NSH2) with paxillin-marked FAs did not change in cells treated with PP2 (Fig. 2b). To further examine the effect of FAK or SRC activity on the association of SRC_SH2 and SHP2_N-SH2 domains with FAs in living cells, we applied time-lapse TIRFM to examine cells that were co-expressing mApple-paxillin and pGFP-C1, mApple-paxillin and pGFP-SRC(SH2) or mApple-paxillin and pGFP-SHP2(NSH2). Analysis of GFP, GFP-SRC(SH2) and GFP-SHP2(NSH2) dynamics during perfusion with FAKi for 1 h revealed that the reduction in FAK activity caused the dissociation of GFP-SRC(SH2), but not GFP or GFP-SRC(NSH2), from paxillin-marked FAs (Fig. 2c). Next we quantified the ratio of fluorescence density of GFP, GFP-SRC(SH2) and GFP-SHP2(NSH2) in paxillin-marked FAs and the results indicated that FAK activity was able to significantly increase the density of GFP-SRC(SH2) in FAs (1.57-fold), but caused no effect on GFP or GFP-SHP2(NSH2) (Fig. 2d). However, treatment with PP2 (treatment with SU6656 was not carried out because it exhibits autofluorescence) did not affect the association of GFP, GFP-SRC(SH2) or GFP-SHP2(NSH2) with paxillin-marked FAs (Fig. 2c,d), which supports the findings presented in Fig. 2b. Thus, the SRC_SH2 domain is able to bring about accumulation in FAs and this event is positively regulated by FAK activity.

**Figure 3:**
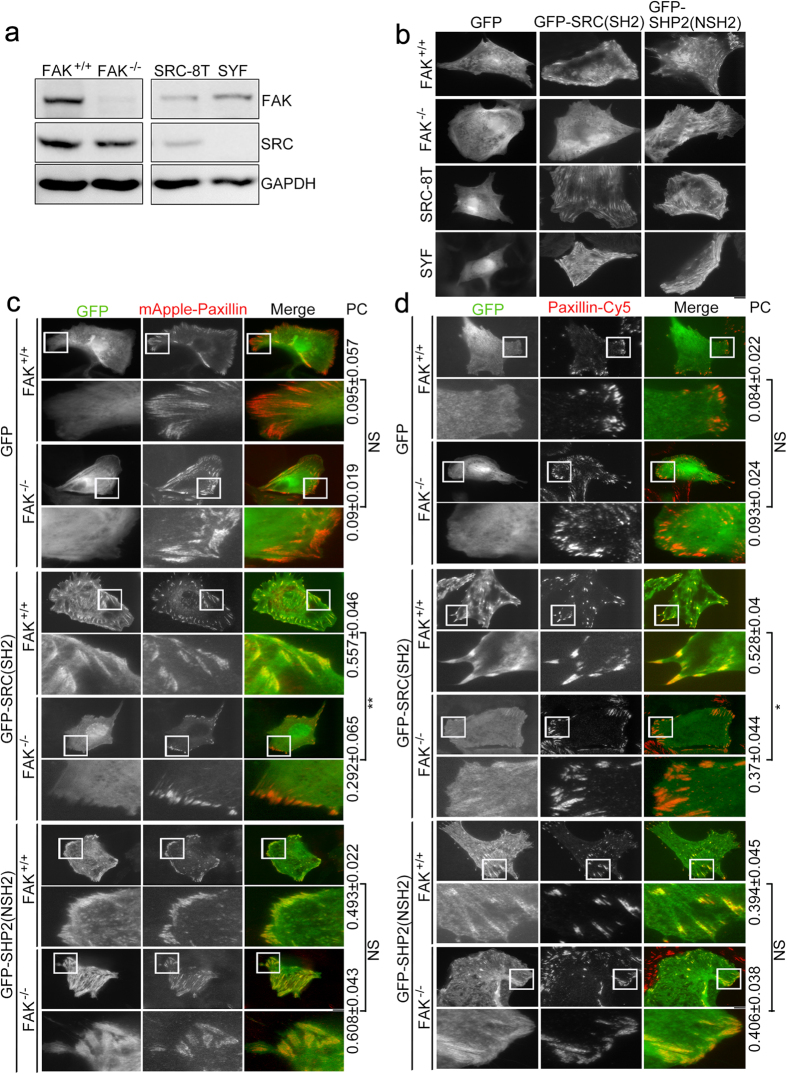
Focal adhesion kinase expression controls the abundance of SRC_SH2 domain within focal adhesions. (**a**) Total cell lysate from FAK^+/+^, FAK^−/−^, SRC-8T (SRC^+/+^/YES^+/+^/FYN^+/+^), and SYF (SRC^−/−^/YES^−/−^/FYN^−/−^) cells were analyzed by Western blotting to detect FAK, SRC and GAPDH. (**b**) TIRFM images of FAK^+/+^, FAK^−/−^, SRC-8T, and SYF cells expressing GFP, GFP-SRC(SH2) or GFP-SHP2(NSH2). Scale bar, 10 μm. (**c**) TIRFM images of FAK^+/+^ and FAK^−/−^ cells co-transfected with mApple-paxillin (red) and pGFP-C1, pGFP-SRC(SH2) or pGFP-SHP2(NSH2) (green). Bar, 10 μm. The 20 μm × 20 μm areas indicated in the upper images are magnified in the images below. Values indicate Pearson’s correlation coefficients (PC) of images of mApple-paxillin and GFP, GFP-SRC(SH2) or GFP-SHP2(NSH2) in FAK^+/+^ or FAK^−/−^ cells. Data are means ± s.e.m. (GFP: n = 3 FAK^+/+^cells, n = 4 FAK^−/−^ cells; GFP-SRC(SH2): n = 5 FAK^+/+^cells, n = 5 FAK^−/−^ cells; GFP-SHP2(NSH2): n = 3 FAK^+/+^cells, n 9 FAK^−/−^ cells). **p < 0.01; NS, no significance. (**d**) TIRFM images of FAK^+/+^ and FAK^−/−^ cells transfected with pGFP-C1, pGFP-SRC(SH2) or pGFP-SHP2(NSH2) (green) and immunostained for paxillin (red). Bar, 10 μm. The 20 μm × 20 μm areas indicated in the upper images are magnified in the images below. Values indicate PC of images of paxillin-Cy5 and GFP, GFP-SRC(SH2) or GFP-SHP2(NSH2) in FAK^+/+^ or FAK^−/−^ cells. Data are means ± s.e.m. (GFP: n = 4 FAK^+/+^cells, n = 3 FAK^−/−^ cells; GFP-SRC(SH2): n = 6 FAK^+/+^cells, n = 4 FAK^−/−^ cells; GFP-SHP2(NSH2): n = 6 FAK^+/+^cells, n = 8 FAK^−/−^ cells). *p < 0.05; NS, no significance.

To further determine whether expression of FAK or expression of SRC regulates the association of SRC_SH2 domain or SHP2_N-SH2 domain with FAs, we visualized the localization of GFP-SRC(SH2) and GFP-SHP2(NSH2) in FAK^+/+^, FAK^−/−^, SRC-8T (SRC^+/+^/YES^+/+^/FYN^+/+^) and SYF (SRC^−/−^/YES^−/−^/FYN^−/−^) MEF cells (Fig. 3a). TIRFM images of the cells expressing pGFP-C1, pGFP-SRC(SH2) or pGFP-SHP2(NSH2) showed that GFP-SRC(SH2) seemed to be able to localize at FAs in FAK^+/+^, SRC-8T and SYF cells, but not in FAK^−/−^ cells. However, GFP-SHP2(NSH2) seemed to be localized at FAs in all of these different cell types (Fig. 3b). To further confirm whether the FA localization of the SRC_SH2 domain and the SHP2_N-SH2 domain occurs in response to FAK or SRC expression, pGFP-C1, pGFP-SRC(SH2) or pGFP-SHP2(NSH2) were co-expressed with mApple-paxillin in FAK^+/+^, FAK^−/−^, SRC-8T and SYF cells. TIRFM imaging revealed the absence of GFP-SRC(SH2) at paxillin-marked FAs in FAK^−/−^ cells, compared with FAK^+/+^cells (Fig. 3c), SRC-8T and SYF cells (Supplemental Fig. 2a). On the other hand, GFP-SHP2(NSH2) did co-localize with mApple-paxillin at FAs in FAK^+/+^, FAK^−/−^, SRC-8T and SYF cells (Fig. 3c and Supplemental Fig. 2a). Immunostaining of cells expressing pGFP-C1, pGFP-SRC(SH2) or pGFP-SHP2(NSH2) with paxillin antibodies tagged with Cy5 also revealed similar results as shown in Fig. 3c and Supplemental Fig. 2a (Fig. 3d and Supplemental Fig. 2b). Thus, the SRC_SH2 domain is able to bring about the concentration of this protein within FAs and this event occurs in a FAK-dependent manner.

### The association of SRC with FAs depends mainly on its SH2 domain, which is regulated by FAK

**Figure 4:**
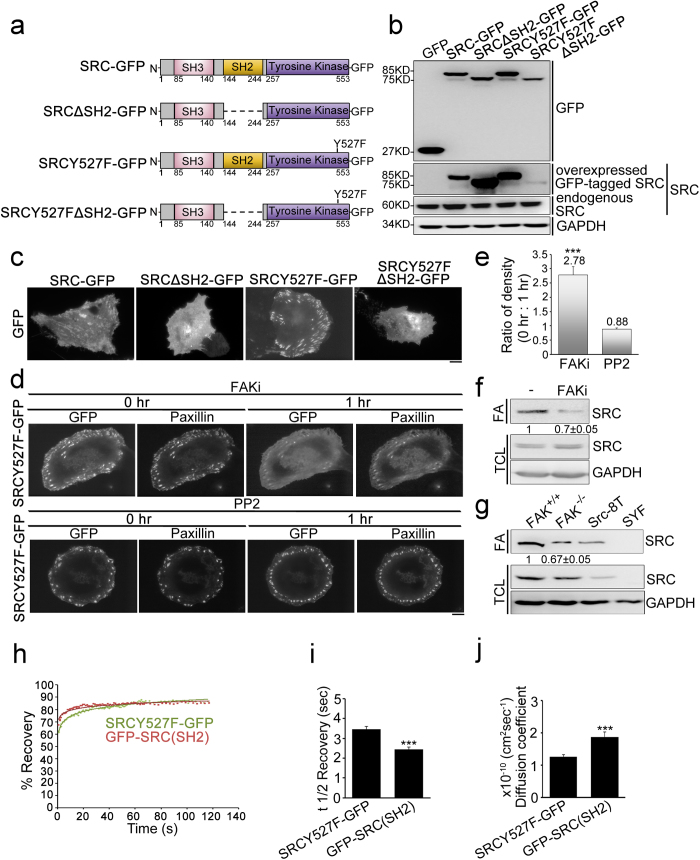
The localization of SRC at focal adhesions depends on its SH2 domain. (**a**) Diagram of the domain structures of SRC-GFP, SRCΔSH2-GFP, SRCY527F-GFP and SRCY527FΔSH2-GFP. (**b**) Cell lysates from U2OS cell expressing GFP, SRC-GFP, SRCΔSH2-GFP, SRCY527F-GFP or SRCY527FΔSH2-GFP were analyzed by Western blotting. (**c**) TIRFM images of U2OS cells expressing SRC-GFP, SRCΔSH2-GFP, SRCY527F-GFP or SRCY527FΔSH2-GFP. Scale bar, 10 μm. (**d**) Time-lapse TIRFM images of U2OS cells co-transfected with mApple-paxillin (red) and SRCY527F-GFP (green) that have been treated with FAKi or PP2. Time is shown in hours. Bar, 10 μm. (**e**) The ratio of the average density (intensity per μm^2^) of SRCY527F-GFP within segmented paxillin-marked FAs within U2OS treated with FAKi or PP2 for 0 h relative to 1 h. Data are means ± s.e.m. (FAKi: n = 11 cells; PP2: n = 14 cells). ***p < 0.001. (**f**) Total cell lysate (TCL) and the focal adhesion fraction (FA) from U2OS cells untreated or treated with FAKi were analyzed by Western blotting for SRC and GAPDH. Data are mean ± s.e.m. (n = 3 individual experiments). (**g**) Total cell lysate (TCL) and focal adhesion fraction (FA) from FAK^+/+^, FAK^−/−^, SRC-8T, and SYF cells were analyzed by Western blotting for SRC and GAPDH. Data are means ± s.e.m. (n = 3 individual experiments). (h, i, j) SRCY527F-GFP and GFP-SRC(SH2) localized to FAs were subjected to FRAP: (**h**) Sample fluorescence recovery curves for SRCY527F-GFP and GFP-SRC(SH2) within a single FA. (**i**) Half-times of fluorescence recovery. (**j**) Diffusion coefficient of fluorescence recovery. Data are means ± s.e.m. (SRCY527F-GFP: n = 78 FAs/12 cells; GFP-SRC(SH2): n = 54 FAs/16 cells). ***p < 0.001.

We next sought to examine whether the SRC_SH2 domain is required for the association of SRC with FAs, since the mechanism controlling SRC FA targeting is still controversial. We generated a series of mutants of chicken c-SRC that were GFP tagged at the C terminus, namely SRC-GFP (wild-type), SRCΔSH2-GFP (wild-type SRC without SH2 domain), SRCY527F-GFP (open-conformation constitutively active SRC) and SRCY527FΔSH2-GFP (open-conformation constitutively active SRC without SH2 domain) (Fig. 4a,b). These GFP-tagged SRC mutants were expressed in U2OS cells and imaged by TIRFM. SRC-GFP and SRCY527F-GFP displayed membrane association, while SRCY527F-GFP showed significant localization within FAs, reflecting the association of activated SRC with FAs (Fig. 4c). By way of contrast, SRCY527FΔSH2-GFP was not present within FAs, indicating that SH2 domain is required to active SRC FA targeting.

The SRC_SH2 domain has been demonstrated to associate with FAs in FAK-dependent manner (Figs 2,3) and therefore it is important to determine the effect of FAK on the association of active SRC with FAs in living cells. After treatment with FAKi, it was found that the expression levels of GFP, SRC-GFP, SRC∆SH2-GFP, SRCY527F-GFP and SRCY527F∆SH2-GFP were not changed (Supplemental Fig. 3). Time-lapse TIRFM imaging (at 1 h intervals) of cells expressing SRCY527F-GFP and mApple-paxillin after treatment of FAKi showed that SRCY527F-GFP exhibited an association with paxillin-marked FAs in cells before treatment (at the 0 h time point), followed by a dissociation from the paxillin-marked FAs at the 1 h time point after FAKi treatment (Fig. 4d). A quantification of the fluorescence density of SRCY527F-GFP within FAs indicated that FAK activity was able to significantly increase the FA density of SRCY527F-GFP (by 2.78-fold) (Fig. 4e). On the other hand, when cells were treated with PP2, SRCY527F-GFP colocalization to paxillin-marked FAs at 1 h time point was similar to that of the control at 0 h time point (Fig. 4d,e). Therefore, the activity of FAK and not that of the SRC-family kinases in general is able to enhance the concentration of SRCY527F in FAs. Next, in order to determine whether endogenous SRC accumulation in FAs is regulated by FAK, we used FA isolation^8^,^47^ in order to biochemically isolate the FA fraction from U2OS cells that had or had not been treated with FAKi (Fig. 4f); specially, we examined the abundance of SRC by Western blot analysis. Our results showed that an inhibition of FAK activity resulted in a decrease in the presence of endogenous SRC in the FA fraction by 0.7-fold compared to the control (Fig. 4f). Similarity, in FAK^−/−^ cells, the abundance of SRC in the FA fractions was significantly decreased by 0.67-fold compared to FAK^+/+^ cells (Fig. 4g). Thus, our analysis of the presence of SRC in various isolated FA fractions confirmed that the concentration of SRC in FAs appeared to be positively regulated by FAK activity and FAK expression.

Our findings indicate that there is an association of constitutively active SRC (SRCY527F) with FAs via the SH2 domain and therefore we hypothesized that the SRC_SH2 domain and SRCY527F have a similar adhesion binding affinity. To test this, we applied fluorescence recovery after photobleaching (FRAP) to SRCY527F-GFP and GFP-SRC(SH2) separately using individual FAs as the target (Fig. 4h). We then calculated the mobile and immobile fractions in order to provide a measure of the extent to which the GFP-tagged proteins are able to move within FAs. GFP-SRC(SH2) protein (83.15 ± 1.33%) appeared to be significantly more mobile than SRCY527F-GFP protein (79.73 ± 0.92%) (Supplemental Fig. 4) (χ^2^ = 10.39, *p* = 0.0156; see Supplemental Table 3). In addition, we calculated the mean fluorescence recovery time *t*_1/2_ and the diffusion coefficient in order to assess the stability of FA binding. GFP-SRC(SH2) protein had a shorter FRAP *t*_1/2_ (2.44 ± 0.11 sec) and a higher diffusion coefficient [(1.88 ± 0.15) × 10^−10^ cm^2^/sec] than SRCY527F-GFP protein [FRAP *t*_1/2_: 3.47 ± 0.12 sec; diffusion coefficient: (1.26 ± 0.06) × 10^−10^ cm^2^/sec] (Fig. 4i,j). These findings indicate that the SRC_SH2 domain has a higher protein turnover within FAs than SRCY527F, which suggests that constitutively active SRCY527F is also able to interact with slowly diffusing proteins within the FAs via regions other than the SH2 domain.

### The association of SRC with FAs plays a crucial role in regulation of FA-mediated signaling and cell migration

**Figure 5:**
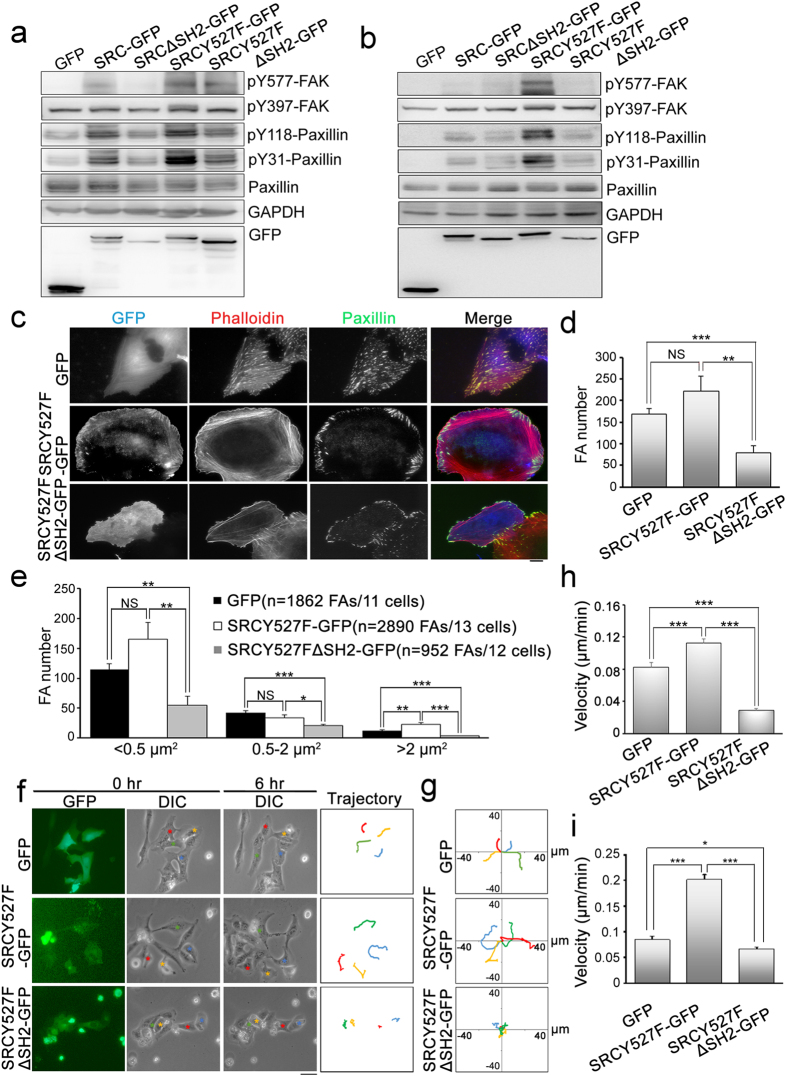
Focal adhesion association of SRC controls SRC-mediated substrate phosphorylation, focal adhesion formation and cell migration. (**a**) Cell lysates from U2OS cells expressing GFP, SRC-GFP, SRCΔSH2-GFP, SRCY527F-GFP or SRCY527FΔSH2-GFP were analyzed by Western blotting for pY577-FAK, pY397-FAK, pY118-paxillin, pY31-paxillin, paxillin, GAPDH and GFP. (**b**) Cell lysates from SYF cells expressing GFP, SRC-GFP, SRCΔSH2-GFP, SRCY527F-GFP or SRCY527FΔSH2-GFP were analyzed by Western blotting for pY577-FAK, pY397-FAK, pY118-paxillin, pY31-paxillin, paxillin, GAPDH and GFP. (**c**) U2OS cells expressing GFP, SRCY527F-GFP or SRCY527FΔSH2-GFP (blue) were immunostained to detect F-actin (phalloidin; red) and paxillin (green), and imaged by epi-fluorescence and TIRFM, respectively. Scale bar, 10 μm. (**d**) The number of segmented paxillin-marked FAs within U2OS cells, as described in (**c**). Data are means ± s.e.m. (GFP: n = 11 cells; SRCY527F-GFP: n = 13 cells; SRCY527FΔSH2-GFP: n = 12 cells). **p < 0.01; ***p < 0.001; NS, no significance. (**e**) Size distribution of segmented paxillin-marked FAs of U2OS cells, as described in (**c**). Data are means ± s.e.m. *p < 0.05; **p < 0.01; ***p < 0.001; NS, no significance. (**f**) The migratory behavior of U2OS cells expressing GFP, SRCY527F-GFP or SRCY527FΔSH2-GFP for 6 hrs. Cells were plated for 16 hrs, and then monitored for 6 hrs. (right) These images delineate the trajectory of the GFP-marked cells over a 6-hr period. Colored stars and lines were used to distinguish cell trajectories. Scale bar, 50 μm. (**g**) Analysis of migration trajectories. The trajectories of representative cells are plotted. The origins of migration are superimposed at (0, 0). (**h**) Migration velocity was calculated as described in Methods. Data are means ± s.e.m. (GFP: n = 58 cells from 3 individual experiments; SRCY527F-GFP: n = 65 cells from individual experiments; SRCY527FΔSH2-GFP: n = 58 cells from 3 individual experiments). ***p < 0.001. (**i**) Migration velocity of SRC-8T (SRC^+/+^/YES^+/+^/FYN^+/+^) MEFs expressing GFP, SRCY527F-GFP or SRCY527FΔSH2-GFP within 6 hrs were calculated as described in Methods. Data are means ± s.e.m. (GFP: n = 28 cells from 3 individual experiments; SRCY527F-GFP: n = 120 cells from 3 individual experiments; SRCY527FΔSH2-GFP: n = 91 cells from individual experiments). *p < 0.05; ***p < 0.001.

Although SRC seems to be able to interact with proteins within FAs using regions other than the SH2 domain, the SH2 domain was confirmed to play a crucial role in constitutively active SRC FA targeting (Fig. 4c). Therefore, we focused our investigation on the association of SRC with the functioning of FAs. First we determined whether the SRC_SH2 domain is required for SRC-mediated substrate phosphorylation, including the formation of pY31-paxillin, pY118-paxillin and pY577-FAK^46^,^48^; this was carried out in either U2OS or SYF cells. Cell lysates were immunoblotted with phosphospecific antibodies and the results showed that, in U2OS cells, SRC-GFP and SRCY527F-GFP independently were able to significantly enhance the levels of pY31-paxillin, pY118-paxillin and pY577-FAK compared with cells expressing only GFP. Separately, in lysates of either SRC∆SH2-GFP or SRCY527F∆SH2-GFP transfected cells, the levels of pY31-paxillin, pY118-paxillin and pY577-FAK were substantially reduced compared to cells expressing SRC-GFP or SRCY527F-GFP, respectively (Fig. 5a). In SYF cells, the levels of pY31-paxillin, pY118-paxillin and pY577-FAK were significantly enhanced when SRCY527F-GFP was expressed but not when SRCY527FΔSH2-GFP was expressed (Fig. 5b). These findings indicate that SRC FA targeting via its SH2 domain is required for SRC-mediated substrate phosphorylation.

Paxillin phosphorylation is known to modify FA dynamics and cell migration^49^,^50^,^51^,^52^,^53^,^54^ and therefore we next explored the effect of SRC FA targeting on FA formation. Immunolocalization of paxillin in cells expressing GFP, SRCY527F-GFP or SRCY527FΔSH2-GFP showed that expression of SRCY527FΔSH2 was able to significantly decrease the total number of FAs present in U2OS cells compared to GFP-expressing or SRCY527F-expressing cells (Fig. 5c,d). A quantitative analysis of these paxillin-marked FAs indicated that SRCY527F expression significantly increased the number of large FAs (>2 μm^2^) compared to GFP-expressing cells, while expression of SRCY527FΔSH2 was found to decrease significantly the number of large FAs (>2 μm^2^), medium-sized FAs (0.5 ~ 2 μm^2^) and small FAs (<0.5 μm^2^) compared with GFP or SRCY527F-expressing cells (Fig. 5e). Thus, the association of constitutively active SRC (SRCY527F) with FAs via its SH2 domain appears to modulate SRC-induced FA formation and FA maturation. In addition, SRCY527FΔSH2 expression also plays a dominant-negative role in the suppression of FA formation and FA maturation in U2OS cells.

As the expression of SRC is positively correlated with integrin-mediated cell motility^29^, we next investigated the effect of SRC on migratory behavior and whether this involves FA recruitment of SRC. U2OS cells expressing GFP, SRCY527F-GFP or SRCY527FΔSH2-GFP were plated on fibronectin-coated plates and subjected to random migration analysis. We first analyzed the trajectory of each individual GFP-labeled cell over a 6-hour migration period by tracking the geometric center of each GFP-labeled cell’s nucleus using time-lapse imaging. It was found that, in comparison with GFP-expressing cells, SRCY527F-GFP-expressing cells displayed much longer paths and a greater net translocation, whereas cells expressing SRCY527FΔSH2 showed much shorter paths and lower net migration (Fig. 5f). These findings are more clearly visualized when presented as trajectories on window plots (Fig. 5g). We also calculated the migration velocity (Fig. 5g) and speed (data not shown) of these cells, which revealed that cells expressing SRCY527F showed a significant increase in migration velocity and speed, but that this effect of SRCY527F was blocked by deletion of the SH2 domain (SRCY527FΔSH2). Furthermore, expression of SRCY527F∆SH2 displayed a dominant-negative functionality when suppressing endogenous SRC-mediated cell migration. The same result appeared in SRC-8T MEFs (Fig. 5i). These findings reveal that various types of cell show significant differences in their migration speed and velocity and that these factors may dictate their migration capability. Taken together, our findings suggest that SRC promotes random migration through the recruitment of SRC to FAs via the protein’s SH2 domain and that this leads to a transduction of appropriated SRC-mediated adhesion signals, which in turn control FA formation and FA maturation; these processes thereby enhance cell migration.

## Discussion

We screened the SH2 domains of the FA tyrosine-specific kinases and phosphoatases, including SRC, SHP1 and SHP2 in order to explore the association of the SRC_SH2 domain and the SHP2_N-SH2 domain with FAs. We found that the association of the SRC_SH2 domain with FAs was promoted by the activity of FAK, but not SRC-family kinases; this however was not true for the SHP2_N-SH2 domain. Experiments using deletion of the SH2 domain from a constitutively active SRC (SRCY527F) demonstrated that the SH2 domain was necessary and sufficient for the recruitment of SRCY527F into FAs, and that the recruitment of SRC or SRCY527F into FAs was postively regulated by FAK, but not by SRC-family kinases. This study shows for the first time that the SRC_SH2 domain is necessary and sufficient for SRC specific association with FAs and that the SRC_SH2 domain is a critical factor in SRC signal transduction, FA formation, FA maturation, and cellular migration.

The discovery that the SH2 domain is indispensable for SRC FA targeting is surprising, because both the SH2 domain and the SH3 domain have been considered to act as SRC protein interaction modules^55^ that are able to direct SRC transiently to FAs. In cells expressing SRC-family kinases, a point mutation in the SH2 domain (R175L) of SRC251 (with a deletion mutation in kinase domain of SRC to avoid intramolecular interaction) still allows localization to within FAs^12^, which suggests that there may be a contribution of the SH3 domain or some other residues within the SH2 domain to SRC FA recruitment. In spite of the fact that in SYF cells (SRC^−/−^, Yes^−/−^, Fyn^−/−^) SRC251T175L does not significantly accumulate within FAs, the protein is still able to promote significantly the phosphorylation status of FAK (Y576), paxillin (total pY) and p130Cas (total pY)^39^. This raises the question as to whether the SH2 domain mediates SRC FA targeting producing SRC-mediated signal transduction. Our findings answer this point because we have shown that the SH2 domain is necessary (Fig. 4c) and sufficient (Fig. 1e,f) for recruitment of SRC to FAs, and that the association of SRC with FAs is required for the downstream phosphorylation of various substrates, including pY31-paxillin, pY118-paxillin and pY577-FAK; this is true for both U2OS cells (containing wild-type SRC-family kinases) (Fig. 5a) and SYF cells (SRC^−/−^, Yes^−/−^, Fyn^−/−^) (Fig. 5b). Although the SH3 domain and the N-myristoylation site may contribute to the association of SRC with FAs based on the fact that there is slower diffusion of SRCY527F than the SH2 domain within FAs by FRAP analysis (Fig. 4h–j, Supplemental Fig. 4), we have been able to further confirm that the association of SRCY527F with FAs via its SH2 domain is required for the regulation of FA formation and FA maturation (Fig. 5c–e) and thus the enhancement of cell migration (Fig. 5f–i).

A question remains as to how SRC protein diffusion within FAs is regulated. We have confirmed that the association of SRC with FAs via the protein’s SH2 domain is regulated by FAK (Fig. 4c–g), which is in accord with the report that the mobility-restricting interactions of SRC are largely mediated by its SH2 domain^56^. However, the slower protein turnover of SRCY527F than the SH2 domain within FAs (Fig. 4h,j, Supplemental Fig. 4) indicates that the regions other than the SH2 domain contribute to its mobility-restricting interactions with FA-associated proteins. Some FA-associated protein partners for SRC, such as p130Cas^26^, FAK^27^,^28^ and paxillin^57^, contain both SH2- and SH3-binding sequences, which are able to contribute to SRC binding via the protein’s SH3 domain. Furthermore, a signal sequence for myristoylation at SRC’s N-terminus had been demonstrated to localize SRC to the cell membrane; this may correlate with the mobility-retarding interactions of SRCY527F by its binding to membrane-associated protein substrates. Thus, the SRC domains are responsible for the mobility-retarding interactions of SRCY527F within FAs and whether these domains are involved in the regulation of SRC signal transduction remains to be determined.

Our results have also revealed a previous unrecognized role of the SHP2_N-SH2 domain (5-103 a.a in human), not the SHP2_C-SH2 domain (111-218 a.a in human), namely that the SHP2_N-SH2 domain is sufficient for FA recruitment (Fig. 1d–f). Together with previous studies showing that 46-110 a.a of the mouse SHP2 (*PTPN11* exon 3; 45-109 a.a in human) is required for SHP2 FA targeting^37^, these results indicate that the N-SH2 domain mediates SHP2 recruitment into FAs. However, how N-SH2 domain targets SHP2 to FAs remains an open question. We found that FAK and SRC-family kinases are not able to change the abundance of SHP2_N-SH2 domain protein within FAs (Fig. 2b–d), which implies that FA recruitment of SHP2 occurs in a FAK-independent and SRC-independent manner. Whether SHP2 FA targeting is regulated by N-SH2-associated proteins remains to be determined.

Several questions remain concerning the recruitment of SHP1 to FAs. In spite of the fact that it has been listed in the integrin adhesome^7^, the detailed mechanism of SHP1 FA targeting remains unknown. Our findings have revealed a diffused pattern of the SHP1_SH2 domain proteins (N-SH2 and C-SH2) within the cell cytosol, which indicates that the SH2 domains do not act to direct SHP1 to FAs. Therefore, it is clear that the recruitment of SHP1 to FAs is likely to be mediated through interacting proteins that are yet to be determined.

## Methods

### Cells

U2OS (human bone osteosarcoma cell line), FAK^+/+^ MEFs, FAK^−/−^ MEFs^58^, SRC-8T MEFs and SYF MEFs^29^ were gifts from Prof. R.-H. Chen’s laboratory (Academia Sinica, Taipei, Taiwan), and were maintained in DMEM-high glucose (Invitrogen) supplemented with 10% FBS (Invitrogen) and 1% antibiotics solution (penicillin and streptomycin; Invitrogen) under 5% CO_2_. Transfection was carried out using Lipofectamine 2000 (Invitrogen). For all experiments, cells were seeded on 10 μg/ml fibronectin (homo)-coated coverslips or plates.

### Plasmids, reagents and antibodies

In order to create pGFP-SRC(SH2), pGFP-SHP1(NSH2), pGFP-SHP1(CSH2), pGFP-SHP2(NSH2), and pGFP-SHP2(CSH2), cDNAs encoding the SH2 domains of pBabe-SRCY527F (chicken) (a gift from Prof. R.-H. Chen’s laboratory), pCMV-flag-SHP1 (human), and pCMV-flag-SHP2 (human) (gifts from Prof. Z.-F. Chang’s laboratory, NYMU, Taiwan) were generated by PCR and inserted into pGFP-C1 (Clontech). To create SRCY527F-GFP, full-length SRCY527F cDNA was PCR amplified from the template pBabe-SRCY527F, and cloned into pGFP-N1 (Clontech). To create SRC-GFP, site-directed mutagenesis was performed by amplifying full-length SRC cDNA followed by cloning into pGFP-N1. To create SRCΔSH2-GFP and SRCY527FΔSH2-GFP, the regions SRC_1–144_ and SRC_244–533_ were amplified from SRC-GFP and SRCY527F-GFP, respectively, and cloned into pGFP-N1. The detailed information on the primers use is provided in Supplemental Table 2.

The reagents used were PP2 from Biosource, SU6656 from Biosource and FAK inhibitor from TOCRIS.

The sources of the antibodies used and their dilutions were as follows: mouse anti-paxillin (BD Bioscience 610052; dilution for Western blotting: 1/10000; dilution for immunofluorescence: 1/1000); rabbit anti-paxillin (Santa Cruz Sc-5574; dilution for immunofluorescence: 1/50); rabbit anti-paxillin-pY31 (Invitrogen 44-720G; dilution for Western blotting: 1/1000); rabbit-anti-paxillin-pY118 (Invitrogen 44-722G); dilution for Western blotting: 1/1000); mouse-anti-GAPDH (GeneTex GTX627408; dilution for Western blotting: 1/1000); mouse-anti-GFP (GeneTex GTX628528; dilution for Western blotting: 1/5000); Alexa Fluor 568 phalloidin (Invitrogen A12380; dilution for immunofluorescence: 1/300); rabbit-anti-SRC (Cell Signaling 2108; dilution for Western blotting: 1/1000); rabbit-anti-FAK (Invirtogen AHO0502; dilution for Western blotting: 1/1000); rabbit-anti-FAK-pY397 (Invitrogen 44-625G); dilution for Western blotting: 1/500); rabbit-anti-FAK-pY577 (Invitrogen 44-614ZG); dilution for Western blotting: 1/500); Alexa Fluor 647-anti-mouse IgG (Invitrogen A21236; dilution for immunofluorescence: 1/300); Alexa Fluor 568 phalloidin (Invitrogen 12380; dilution for immunofluorescence: 1/300).

### Immunofluorescence staining and image analysis

For paxillin and phalloidin staining, cells were fixed and immunostained using a method described previously^8^. For TIRFM imaging, cells were mounted on slides with PBS containing N-propyl gallate. TIRFM images were obtained with a 100X, 1.49NA (Oil-Immersion) Plan objective lens (Nikon) using the *iLas* multi-modal of TIRF (Roper)/spinning disk confocal (CSUX1, Yokogawa) microscope system equipped with a Coolsnap HQ2 CCD camera (Photometrics).

The association of GFP-SRC(SH2), GFP-SHP2(NSH2), and SRCY527F-GFP with paxillin-marked FAs was quantified based on the fluorescence intensities of the TIRFM images using Image J to obtain Pearson’s correlation coefficients^59^.

To determine the number of FAs, TIRFM images of paxillin-stained cells were thresholded to highlight only FA and the areas of these regions recorded using Metamorph^8^. The total number of recorded FAs was counted as the total number of FAs. Based on the recorded FA areas, FAs were classified into three size categories, including large (>2 μm^2^), medium-sized (0.5–2 μm^2^), and small (<0.5 μm^2^). The number of large FAs, medium-sized FAs and small FAs was counted individually. The results are presented graphically using Excel software (Microsoft).

### Time-lapse TIRFM

U2OS cells expressing GFP, GFP-SRC(SH2), GFP-SHP2(NSH2) or SRCY527F-GFP together with mApple-paxillin were imaged by dual-color time-lapse TIRFM using the *iLas* multi-modal of TIRF (Roper)/spinning disk confocal (CSUX1, Yokogawa) microscope system. This was equipped with a 100 × 1.49NA (Oil-Immersion) Plan objective lens (Nikon). The TIRFM images were captured using a Coolsnap HQ2 CCD camera (Photometrics).

The relative abundance of GFP-SRC(SH2), GFP-SHP2(NSH2), and SRCY527F-GFP in paxillin-marked FAs was calculated using Metamorph as described previously^8^.

### Random migration analysis

U2OS and SRC-8T cells expressing GFP, SRCY527F-GFP or SRCY527FΔSH2-GFP were plated on six-well plates in the regular culture medium for 16 hrs and then placed in a temperature-controlled and CO_2_-controlled chamber, which was then examined using a microscope (Axio Observer.Z1; Zeiss). Time-Lapse images were obtained at 10 min intervals over 6 hrs using a 10 × 0.25NA objective lens (Zeiss) and an AxioCamMR3 CCD camera. To capture the motility parameters, including velocity and speed, the trajectories of the GFP-labeled cells were recorded by tracing the geometric center of individual GFP-labeled cell’s nucleus for each frame using Metamorph image analysis software (Molecular Device). The results are presented graphically using Excel software (Microsoft). The velocity was calculated as the ratio of the net distance moved (*i.e.* the length of the displacement vector from the initial point to the end-point) and time duration of 6 hr for the period of migration. The speed was determined as the ratio of the total length of the trajectory (the accumulated distance) and time duration, namely 6 hr.

### Fluorescence recovery after photobleaching (FRAP)

U2OS cells were transfected so that they transiently expressed mApple-paxillin in order to localize FAs together with GFP-SRC(SH2) or SRCY527F-GFP. FRAP of GFP-SRC(SH2) or GFP-SRCY527F was performed using a 100 × 1.49NA Plan objective lens on the *iLas* multi-modal of TIRF (Roper)/spinning disk confocal (CSUX1, Yokogawa) microscope system. A 488-nm laser was used to photobleach a spot within an individual fluorescent FA. Images were acquired at 1s intervals before and after the photobleaching using a Coolsnap HQ2 CCD (Photometrics). Image frequency was adjusted depending on the fluorescence photobleaching recovery rate of the GFP-tagged protein that was being imaged. The half-times and the diffusion coefficient of fluorescence recovery, the mobile fraction and the immobile fraction were then calculated using a method described previously^60^.

### Statistical analysis

Statistical significance was measured by Student’s t-test for all the experiments and χ^2^ test for Supplemental Table 3.

## Additional Information

**How to cite this article**: Wu, J.-C. *et al.* Focal adhesion kinase-dependent focal adhesion recruitment of SH2 domains directs SRC into focal adhesions to regulate cell adhesion and migration. *Sci. Rep.* **5**, 18476; doi: 10.1038/srep18476 (2015).

## Supplementary Material

Supplementary Information

## References

[b1] Friedl P. & Wolf K. Tumour-cell invasion and migration: diversity and escape mechanisms. Nat Rev Cancer 3, 362–374 (2003). 12724734 10.1038/nrc1075

[b2] Lauffenburger D. A. & Horwitz A. F. Cell migration: a physically integrated molecular process. Cell 84, 359–369 (1996). 8608589 10.1016/s0092-8674(00)81280-5

[b3] Webb D. J., Parsons J. T. & Horwitz A. F. Adhesion assembly, disassembly and turnover in migrating cells – over and over and over again. Nat Cell Biol 4, E97–100 (2002). 11944043 10.1038/ncb0402-e97

[b4] Burridge K., Fath K., Kelly T., Nuckolls G. & Turner C. Focal adhesions: transmembrane junctions between the extracellular matrix and the cytoskeleton. Annu Rev Cell Biol 4, 487–525 (1988). 3058164 10.1146/annurev.cb.04.110188.002415

[b5] Gupton S. L. & Waterman-Storer C. M. Spatiotemporal feedback between actomyosin and focal-adhesion systems optimizes rapid cell migration. Cell 125, 1361–1374 (2006). 16814721 10.1016/j.cell.2006.05.029

[b6] Hynes R. O. Integrins: bidirectional, allosteric signaling machines. Cell 110, 673–687 (2002). 12297042 10.1016/s0092-8674(02)00971-6

[b7] Zaidel-Bar R., Itzkovitz S., Ma’ayan A., Iyengar R. & Geiger B. Functional atlas of the integrin adhesome. Nat Cell Biol 9, 858–867 (2007). 17671451 10.1038/ncb0807-858 PMC2735470

[b8] Kuo J. C., Han X., Hsiao C. T., Yates J. R. 3rd & Waterman C. M. Analysis of the myosin-II-responsive focal adhesion proteome reveals a role for beta-Pix in negative regulation of focal adhesion maturation. Nat Cell Biol 13, 383–393 (2011). 21423176 10.1038/ncb2216 PMC3279191

[b9] Kuo J. C. Mechanotransduction at focal adhesions: integrating cytoskeletal mechanics in migrating cells. J Cell Mol Med 17, 704–712 (2013). 23551528 10.1111/jcmm.12054 PMC3823174

[b10] Kuo J. C. Focal adhesions function as a mechanosensor. Prog Mol Biol Transl Sci 126, 55–73 (2014). 25081614 10.1016/B978-0-12-394624-9.00003-8

[b11] Pawson T., Gish G. D. & Nash P. SH2 domains, interaction modules and cellular wiring. Trends Cell Biol 11, 504–511 (2001). 11719057 10.1016/s0962-8924(01)02154-7

[b12] Kaplan K. B. *et al.* Association of the amino-terminal half of c-Src with focal adhesions alters their properties and is regulated by phosphorylation of tyrosine 527. EMBO J 13, 4745–4756 (1994). 7525268 10.1002/j.1460-2075.1994.tb06800.x PMC395413

[b13] Resh M. D. Fatty acylation of proteins: new insights into membrane targeting of myristoylated and palmitoylated proteins. Biochim Biophys Acta 1451, 1–16 (1999). 10446384 10.1016/s0167-4889(99)00075-0

[b14] Brown M. T. & Cooper J. A. Regulation, substrates and functions of src. Biochim Biophys Acta 1287, 121–149 (1996). 8672527 10.1016/0304-419x(96)00003-0

[b15] Chong Y. P. *et al.* C-terminal Src kinase-homologous kinase (CHK), a unique inhibitor inactivating multiple active conformations of Src family tyrosine kinases. J Biol Chem 281, 32988–32999 (2006). 16959780 10.1074/jbc.M602951200

[b16] Xu W., Harrison S. C. & Eck M. J. Three-dimensional structure of the tyrosine kinase c-Src. Nature 385, 595–602 (1997). 9024657 10.1038/385595a0

[b17] Su J., Muranjan M. & Sap J. Receptor protein tyrosine phosphatase alpha activates Src-family kinases and controls integrin-mediated responses in fibroblasts. Curr Biol 9, 505–511 (1999). 10339427 10.1016/s0960-9822(99)80234-6

[b18] Tonks N. K. & Neel B. G. Combinatorial control of the specificity of protein tyrosine phosphatases. Curr Opin Cell Biol 13, 182–195 (2001). 11248552 10.1016/s0955-0674(00)00196-4

[b19] Zheng X. M., Resnick R. J. & Shalloway D. A phosphotyrosine displacement mechanism for activation of Src by PTPalpha. EMBO J 19, 964–978 (2000). 10698938 10.1093/emboj/19.5.964 PMC305636

[b20] Briggs S. D., Sharkey M., Stevenson M. & Smithgall T. E. SH3-mediated Hck tyrosine kinase activation and fibroblast transformation by the Nef protein of HIV-1. J Biol Chem 272, 17899–17902 (1997). 9218412 10.1074/jbc.272.29.17899

[b21] Chong Y. P., Ia K. K., Mulhern T. D. & Cheng H. C. Endogenous and synthetic inhibitors of the Src-family protein tyrosine kinases. Biochim Biophys Acta 1754, 210–220 (2005). 16198159 10.1016/j.bbapap.2005.07.027

[b22] Courtneidge S. A. Role of Src in signal transduction pathways. The Jubilee Lecture. Biochem Soc Trans 30, 11–17 (2002). 12023816 10.1042/

[b23] Frame M. C. Newest findings on the oldest oncogene; how activated src does it. J Cell Sci 117, 989–998 (2004). 14996930 10.1242/jcs.01111

[b24] Martin G. S. The hunting of the Src. Nat Rev Mol Cell Biol 2, 467–475 (2001). 11389470 10.1038/35073094

[b25] Moarefi I. *et al.* Activation of the Src-family tyrosine kinase Hck by SH3 domain displacement. Nature 385, 650–653 (1997). 9024665 10.1038/385650a0

[b26] Nakamoto T., Sakai R., Ozawa K., Yazaki Y. & Hirai H. Direct binding of C-terminal region of p130Cas to SH2 and SH3 domains of Src kinase. J Biol Chem 271, 8959–8965 (1996). 8621540 10.1074/jbc.271.15.8959

[b27] Thomas J. W. *et al.* SH2- and SH3-mediated interactions between focal adhesion kinase and Src. J Biol Chem 273, 577–583 (1998). 9417118 10.1074/jbc.273.1.577

[b28] Cary L. A., Klinghoffer R. A., Sachsenmaier C. & Cooper J. A. SRC catalytic but not scaffolding function is needed for integrin-regulated tyrosine phosphorylation, cell migration, and cell spreading. Mol Cell Biol 22, 2427–2440 (2002). 11909938 10.1128/MCB.22.8.2427-2440.2002 PMC133722

[b29] Klinghoffer R. A., Sachsenmaier C., Cooper J. A. & Soriano P. Src family kinases are required for integrin but not PDGFR signal transduction. EMBO J 18, 2459–2471 (1999). 10228160 10.1093/emboj/18.9.2459 PMC1171328

[b30] Mayer B. J., Hirai H. & Sakai R. Evidence that SH2 domains promote processive phosphorylation by protein-tyrosine kinases. Curr Biol 5, 296–305 (1995). 7780740 10.1016/s0960-9822(95)00060-1

[b31] Freeman R. M. Jr., Plutzky J. & Neel B. G. Identification of a human src homology 2-containing protein-tyrosine-phosphatase: a putative homolog of Drosophila corkscrew. Proc Natl Acad Sci USA 89, 11239–11243 (1992). 1280823 10.1073/pnas.89.23.11239 PMC50525

[b32] Plutzky J., Neel B. G. & Rosenberg R. D. Isolation of a src homology 2-containing tyrosine phosphatase. Proc Natl Acad Sci USA 89, 1123–1127 (1992). 1736296 10.1073/pnas.89.3.1123 PMC48398

[b33] Shen S. H., Bastien L., Posner B. I. & Chretien P. A protein-tyrosine phosphatase with sequence similarity to the SH2 domain of the protein-tyrosine kinases. Nature 352, 736–739 (1991). 1652101 10.1038/352736a0

[b34] Lin S. Y. *et al.* The protein-tyrosine phosphatase SHP-1 regulates the phosphorylation of alpha-actinin. J Biol Chem 279, 25755–25764 (2004). 15070900 10.1074/jbc.M314175200

[b35] von Wichert G., Haimovich B., Feng G. S. & Sheetz M. P. Force-dependent integrin-cytoskeleton linkage formation requires downregulation of focal complex dynamics by Shp2. EMBO J 22, 5023–5035 (2003). 14517241 10.1093/emboj/cdg492 PMC204475

[b36] Yu D. H., Qu C. K., Henegariu O., Lu X. & Feng G. S. Protein-tyrosine phosphatase Shp-2 regulates cell spreading, migration, and focal adhesion. J Biol Chem 273, 21125–21131 (1998). 9694867 10.1074/jbc.273.33.21125

[b37] Lee H. H. *et al.* Shp2 plays a crucial role in cell structural orientation and force polarity in response to matrix rigidity. Proc Natl Acad Sci USA 110, 2840–2845 (2013). 23359696 10.1073/pnas.1222164110 PMC3581906

[b38] Felsenfeld D. P., Schwartzberg P. L., Venegas A., Tse R. & Sheetz M. P. Selective regulation of integrin--cytoskeleton interactions by the tyrosine kinase Src. Nat Cell Biol 1, 200–206 (1999). 10559917 10.1038/12021

[b39] Yeo M. G. *et al.* Src SH2 arginine 175 is required for cell motility: specific focal adhesion kinase targeting and focal adhesion assembly function. Mol Cell Biol 26, 4399–4409 (2006). 16738308 10.1128/MCB.01147-05 PMC1489135

[b40] Burridge K., Turner C. E. & Romer L. H. Tyrosine phosphorylation of paxillin and pp125FAK accompanies cell adhesion to extracellular matrix: a role in cytoskeletal assembly. J Cell Biol 119, 893–903 (1992). 1385444 10.1083/jcb.119.4.893 PMC2289706

[b41] Guan J. L. & Shalloway D. Regulation of focal adhesion-associated protein tyrosine kinase by both cellular adhesion and oncogenic transformation. Nature 358, 690–692 (1992). 1379699 10.1038/358690a0

[b42] Hanks S. K., Calalb M. B., Harper M. C. & Patel S. K. Focal adhesion protein-tyrosine kinase phosphorylated in response to cell attachment to fibronectin. Proc Natl Acad Sci USA 89, 8487–8491 (1992). 1528852 10.1073/pnas.89.18.8487 PMC49945

[b43] Lipfert L. *et al.* Integrin-dependent phosphorylation and activation of the protein tyrosine kinase pp125FAK in platelets. J Cell Biol 119, 905–912 (1992). 1385445 10.1083/jcb.119.4.905 PMC2289696

[b44] Schaller M. D. *et al.* pp125FAK a structurally distinctive protein-tyrosine kinase associated with focal adhesions. Proc Natl Acad Sci USA 89, 5192–5196 (1992). 1594631 10.1073/pnas.89.11.5192 PMC49256

[b45] Pasapera A. M., Schneider I. C., Rericha E., Schlaepfer D. D. & Waterman C. M. Myosin II activity regulates vinculin recruitment to focal adhesions through FAK-mediated paxillin phosphorylation. J Cell Biol 188, 877–890 (2010). 20308429 10.1083/jcb.200906012 PMC2845065

[b46] Vindis C., Teli T., Cerretti D. P., Turner C. E. & Huynh-Do U. EphB1-mediated cell migration requires the phosphorylation of paxillin at Tyr-31/Tyr-118. J Biol Chem 279, 27965–27970 (2004). 15107421 10.1074/jbc.M401295200

[b47] Kuo J. C., Han X., Yates J. R. 3rd & Waterman C. M. Isolation of focal adhesion proteins for biochemical and proteomic analysis. Methods Mol Biol 757, 297–323 (2012). 21909920 10.1007/978-1-61779-166-6_19 PMC4158431

[b48] Calalb M. B., Polte T. R. & Hanks S. K. Tyrosine phosphorylation of focal adhesion kinase at sites in the catalytic domain regulates kinase activity: a role for Src family kinases. Mol Cell Biol 15, 954–963 (1995). 7529876 10.1128/mcb.15.2.954 PMC231984

[b49] Bertolucci C. M., Guibao C. D. & Zheng J. J. Phosphorylation of paxillin LD4 destabilizes helix formation and inhibits binding to focal adhesion kinase. Biochemistry 47, 548–554 (2008). 18092823 10.1021/bi702103n PMC4054611

[b50] Nayal A. *et al.* Paxillin phosphorylation at Ser273 localizes a GIT1-PIX-PAK complex and regulates adhesion and protrusion dynamics. J Cell Biol 173, 587–589 (2006). 16717130 10.1083/jcb.200509075 PMC2063867

[b51] Petit V. *et al.* Phosphorylation of tyrosine residues 31 and 118 on paxillin regulates cell migration through an association with CRK in NBT-II cells. J Cell Biol 148, 957–970 (2000). 10704446 10.1083/jcb.148.5.957 PMC2174549

[b52] Webb D. J. *et al.* FAK-Src signalling through paxillin, ERK and MLCK regulates adhesion disassembly. Nat Cell Biol 6, 154–161 (2004). 14743221 10.1038/ncb1094

[b53] Zaidel-Bar R., Ballestrem C., Kam Z. & Geiger B. Early molecular events in the assembly of matrix adhesions at the leading edge of migrating cells. J Cell Sci 116, 4605–4613 (2003). 14576354 10.1242/jcs.00792

[b54] Zaidel-Bar R., Milo R., Kam Z. & Geiger B. A paxillin tyrosine phosphorylation switch regulates the assembly and form of cell-matrix adhesions. J Cell Sci 120, 137–148 (2007). 17164291 10.1242/jcs.03314

[b55] Moroco J. A. *et al.* A Discovery Strategy for Selective Inhibitors of c-Src in Complex with the Focal Adhesion Kinase SH3/SH2-binding Region. Chem Biol Drug Des 86, 144–155 (2015). 25376742 10.1111/cbdd.12473 PMC4444405

[b56] Shvartsman D. E. *et al.* Src kinase activity and SH2 domain regulate the dynamics of Src association with lipid and protein targets. J Cell Biol 178, 675–686 (2007). 17698610 10.1083/jcb.200701133 PMC2064473

[b57] Schaller M. D. Paxillin: a focal adhesion-associated adaptor protein. Oncogene 20, 6459–6472 (2001). 11607845 10.1038/sj.onc.1204786

[b58] Ilic D. *et al.* Reduced cell motility and enhanced focal adhesion contact formation in cells from FAK-deficient mice. Nature 377, 539–544 (1995). 7566154 10.1038/377539a0

[b59] Dunn K. W., Kamocka M. M. & McDonald J. H. A practical guide to evaluating colocalization in biological microscopy. Am J Physiol Cell Physiol 300, C723–742 (2011). 21209361 10.1152/ajpcell.00462.2010 PMC3074624

[b60] Lippincott-Schwartz J., Snapp E. & Kenworthy A. Studying protein dynamics in living cells. Nat Rev Mol Cell Biol 2, 444–456 (2001). 11389468 10.1038/35073068

