# Supplementary material for: Focal adhesion kinase-dependent focal adhesion recruitment of SH2 domains directs SRC into focal adhesions to regulate cell adhesion and migration
Source: Sci Rep. 2015 Dec 18;5:18476. doi: 10.1038/srep18476 (PMC4683442; doi:10.1038/srep18476)
Supplement: Supplementary Information [file srep18476-s1.pdf]

Focal adhesion kinase-dependent focal adhesion recruitment of SH2 domains directs SRC into focal adhesions to regulate cell adhesion and migration

Jui-Chung Wu[1], Yu-Chen Chen[1], Chih-Ting Kuo[1], Helen Wenshin Yu[2], Yin-Quan Chen[2], Arthur Chiou[2,3], Jean-Cheng Kuo[1,3,*]

[1] Institute of Biochemistry and Molecular Biology, National Yang-Ming University, Taipei 11221, Taiwan

[2] Institute of Biophotonics, National Yang-Ming University, Taipei 11221, Taiwan

[3] Biophotonics and Molecular Imaging Research Center, National Yang-Ming University, Taipei 11221, Taiwan

[*] Correspondence to: Jean-Cheng Kuo (jckuo@ym.edu.tw)

Supplemental Figure 1

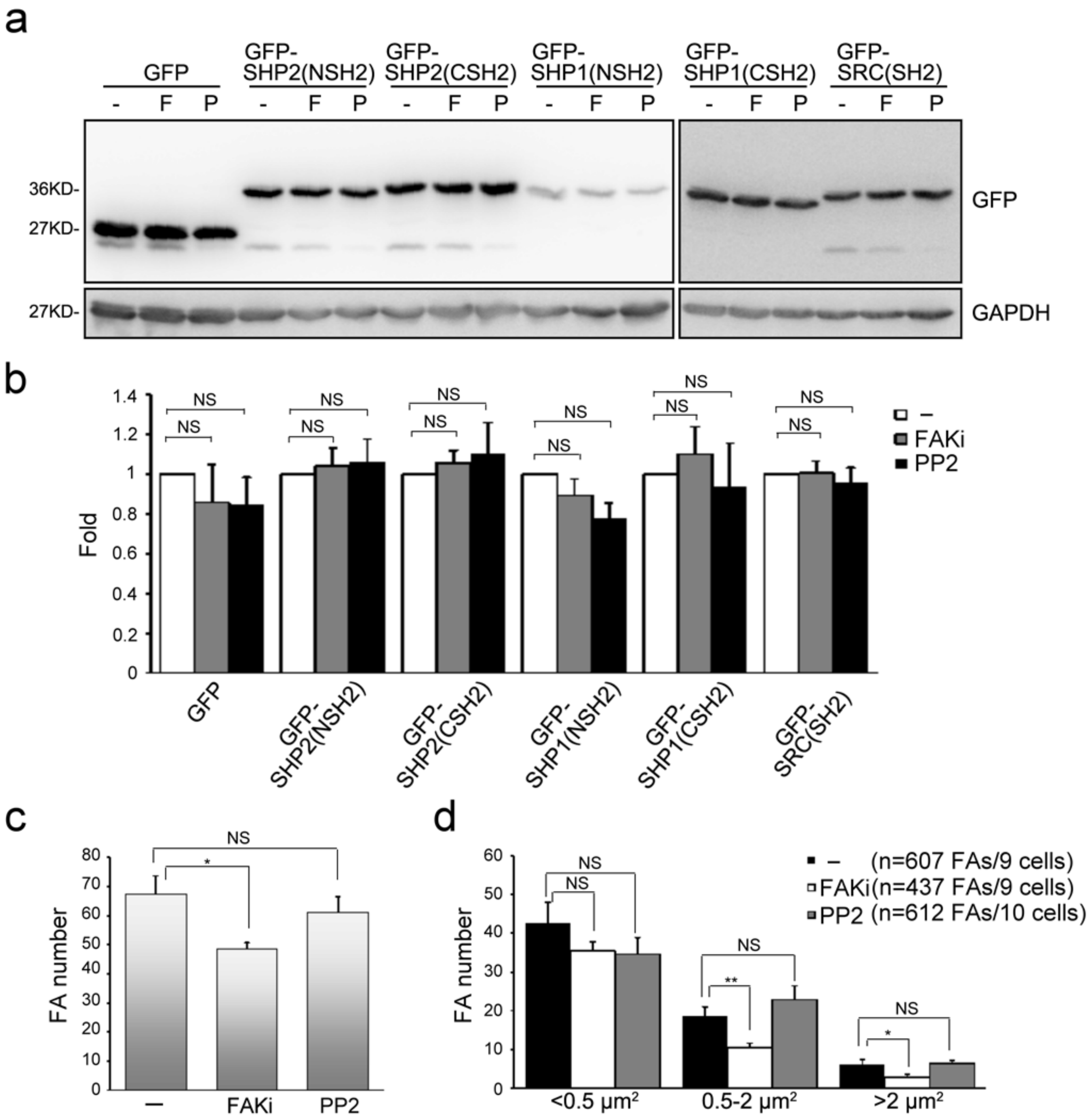

Supplemental Figure 2

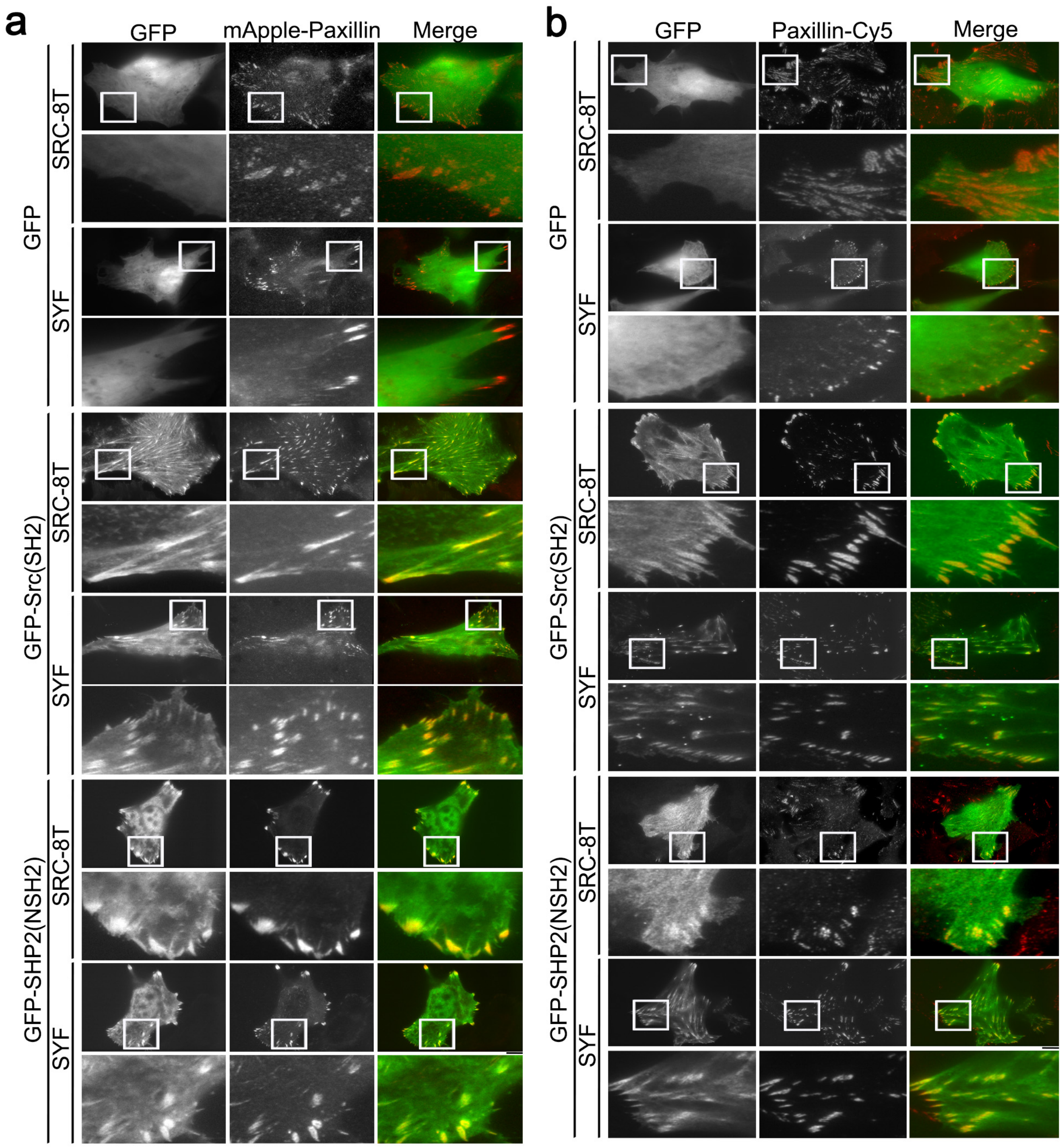

Supplemental Figure 3

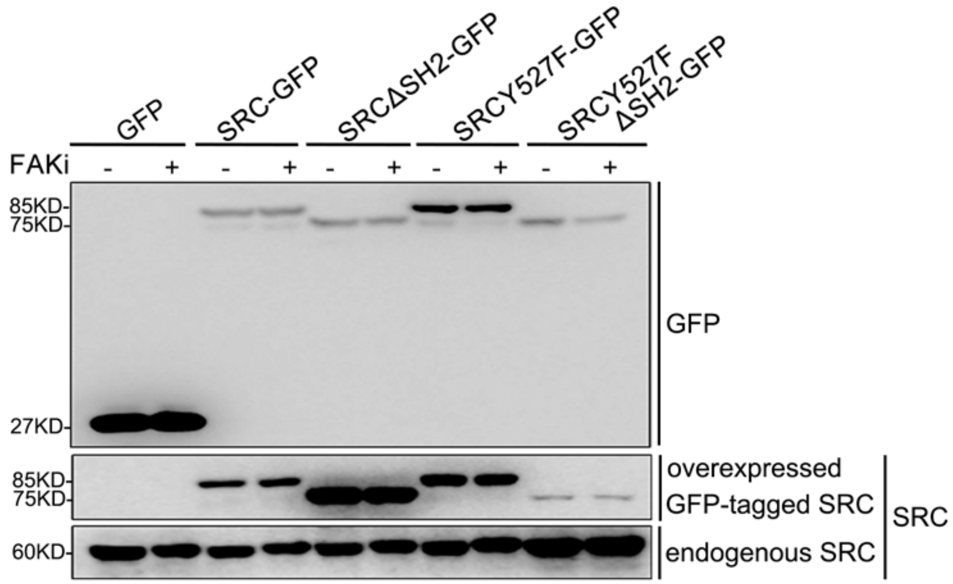

Supplemental Figure 4

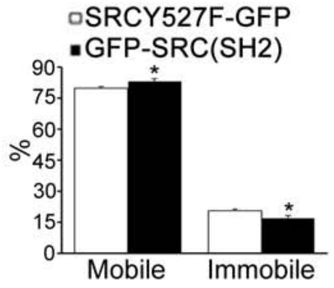

Wu_Supplemental figure legends

Figure S1. The treatment of FAKi or PP2 does not change the expression of GFP-tagged SH2 proteins.

(a) U2OS cells transfected with pGFP-C1, pGFP-SHP2(NSH2), pGFP-SHP2(CSH2), pGFP-SHP1(NSH2), pGFP-SHP1(CSH2) or pGFP-SRC(SH2) were untreated (-) or treated with FAKi (F) or PP2 (P), and analyzed by Western blotting using antibodies specific to GFP or GAPDH. (b) Change relative to control (-) in levels of GFP, GFP-SHP2(NSH2), GFP-SHP2(CSH2), GFP-SHP1(NSH2), GFP-SHP1(CSH2) and GFP-SRC(SH2) were detected by Western blot. Data are means ± s.e.m. (n = 3 individual experiments). NS, no significance. (c) The number of segmented paxillin-stained focal adhesions of U2OS cells untreated (-) or treated with FAKi or PP2. Data are means ± s.e.m. (untreated: n = 9 cells; FAKi: n = 9 cells; PP2: n = 10 cells). *$p < 0.05$; NS, no significance. (d) Size distribution of segmented paxillin-stained focal adhesions of U2OS cells, as described in C. Data are means ± s.e.m. *$p < 0.05$; **$p < 0.01$; NS, no significance.

Figure S2. Inhibition of SRC-family kinases activity does not influence the abundance of GFP, GFP-SRC(SH2) and GFP-SHP2(NSH2) at focal adhesions.

(a) TIRFM images of SRC-8T and SYF cells co-transfected with mApple-paxillin (red) and pGFP-C1, pGFP-SRC(SH2) or pGFP-SHP2(NSH2) (green). Bar, 10 μm. The 20 μm x 20 μm areas indicated in the upper images are magnified in the images below. (b) TIRFM images of SRC-8T and SYF cells transfected with pGFP-C1, pGFP-SRC(SH2) or pGFP-SHP2(NSH2)

(green) and immunostained for paxillin (red). Bar, 10 µm. The 20 µm x 20 µm areas indicated in the upper images are magnified in the images below.

Figure S3. The treatment of FAKi does not change the expression of GFP-tagged SRC proteins.

U2OS cells transfected with GFP, SRC-GFP, SRCΔSH2-GFP, SRCY527F-GFP or SRCY527FΔSH2-GFP were treated with (+) or without (-) FAKi, and analyzed by Western blotting using antibodies specific to GFP or SRC.

Figure S4. SRCY527F protein and SRC_SH2 domain possess different percentage of mobile proportion within focal adhesions.

SRCY527F-GFP and GFP-SRC(SH2) localized to focal adhesions were subjected to FRAP: The percentage of mobile fraction and immobile fraction of SRCY527F-GFP and GFP-SRC(SH2) within focal adhesions. Data are means ± s.e.m. (SRCY527F-GFP: n = 78 focal adhesions / 12 cells; GFP-SRC(SH2): n = 54 focal adhesions / 16 cells). *$p < 0.05$.

Wu_Supplemental Table 1: A list of the cDNA sequences of various SH2 domains.

Name	Sequence
**SHP1(NSH2)**	5’GGTGGTTTCACCGAGACCTCAGTGGGCTGGATGCAGAGACCCT GCTCAAGGGCCGAGGTGTCCACGGTAGCTTCCTGGCTCGGCCCA GTCGCAAGAACCAGGGTGACTTCTCGCTCTCCGTCAGGGTGGGG GATCAGGTGACCCATATTCGGATCCAGAACTCAGGGGATTTCTATG ACCTGTATGGAGGGGAGAAGTTTGCGACTCTGACAGAGCTGGTG GAGTACTACACTCAGCAGCAGGGTGTCCTGCAGGACCGCGACGG CACCATCATCCACCTCAAGTACCCGCTGAAC
**SHP1(CSH2)**	5’AGGTGGTACCATGGCCACATGTCTGGCGGGCAGGCAGAGACGC TGCTGCAGGCCAAGGGCGAGCCCTGGACGTTTCTTGTGCGTGAG AGCCTCAGCCAGCCTGGAGACTTCGTGCTTTCTGTGCTCAGTGAC CAGCCCAAGGCTGGCCCAGGCTCCCCGCTCAGGGTCACCCACAT CAAGGTCATGTGCGAGGGTGGACGCTACACAGTGGGTGGTTTGG AGACCTTCGACAGCCTCACGGACCTGGTGGAGCATTTCAAGAAG ACGGGGATTGAGGAGGCCTCAGGCGCCTTTGTCTACCTGCGGCA GCCGTACTATGCC
**SHP2(NSH2)**	5’ACATCGCGGAGATGGTTTCACCCAAATATCACTGGTGTGGAGGC AGAAAACCTACTGTTGACAAGAGGAGTTGATGGCAGTTTTTTGGC AAGGCCTAGTAAAAGTAACCCTGGAGACTTCACACTTTCCGTTAG AAGAAATGGAGCTGTCACCCACATCAAGATTCAGAACACTGGTG ATTACTATGACCTGTATGGAGGGGAGAAATTTGCCACTTTGGCTG AGTTGGTCCAGTATTACATGGAACATCACGGGCAA
**SHP2(CSH2)**	5’GAAAGGTGGTTTCATGGACATCTCTCTGGGAAAGAAGCAGAGA AATTATTAACTGAAAAAGGAAAACATGGTAGTTTTCTTGTACGAG AGAGCCAGAGCCACCCTGGAGATTTTGTTCTTTCTGTGCGCACTG GTGATGACAAAGGGGAGAGCAATGACGGCAAGTCTAAAGTGACC CATGTTATGATTCGCTGTCAGGAACTGAAATACGACGTTGGTGGA GGAGAACGGTTTGATTCTTTGACAGATCTTGTGGAACATTATAAG AAGAATCCTATGGTG
**Src(SH2)**	5’CAGGCTGAAGAGTGGTACTTTGGGAAGATCACTCGTCGGGAGT CCGAGCGGCTGCTGCTCAACCCCGAAAACCCCCGGGGAACCTTC TTGGTCCGGGAGAGCGAGACGACAAAGGTGCCTATTGCCTCTC CGTTTCTGACTTTGACAACGCCAAGGGGCTCAATGTGAAGCACTA CAAGATCCGCAAGCTGGACAGCGGCGGCTTCTACATCACCTCACG CACACAGTTCAGCAGCCTGCAGCAGCTGGTGGCCTACTACTCCA AACATGCTGATGGCTTGTGCCACCGCCTGACCAACGTC

Wu_Supplemental Table 2: A list of the primers used to create various DNA constructs.

Constructs	Primers
**pGFP-SHP1(NSH2)**	*SHP1-NSH2-F* (HindIII)<br>*GCTCA AGCT*T TGATG AGGTG GTTTC ACCGA GACCT CAGTG<br>*SHP1-NSH2-R* (KpnI)<br>CCGCG GTACC TTAGT TCAGC GGGTA CTTGA GGTGG ATGAT
**pGFP-SHP1(CSH2)**	*SHP1-CSH2-F* (HindIII)<br>GCTCA AGCTT TGATG AGGTG GTACC ATGGC CACAT GTCTG<br>*SHP1-CSH2-R* (BamHI)<br>CGGTG GATCC TTAGG CATAG TACGG CTGCC GCAGG TAGAC
**pGFP-SHP2(NSH2)**	*SHP2-NSH2-F* (KpnI)<br>CGACG GTACC ATGAC ATCGC GGAGA TGGTT TCACC CAAAT<br>*SHP2-NSH2-R* (BamHI)<br>CCCGG GATCC TTATT GCCCG TGATG TTCCA TGTAA TACTG
**pGFP-SHP2(CSH2)**	*SHP2-CSH2-F* (KpnI)<br>CGACG GTACC ATGGA AAGGT GGTTT CATGG ACATC TCTCT<br>*SHP2-CSH2-R* (BamHI)<br>CCCGG GATCC TTACA CCATA GGATT CTTCT TATAA TGTTC
**pGFP-SRC(SH2)**	*Src-SH2-F* (KpnI)<br>CGACG GTACC ATGCA GGCTG AAGAG TGGTA CTTTG GGAAG<br>*Src-SH2-R* (BamHI)<br>CGGTG GATCC TTACA CGTTG GTCAG GCGGT GGCAC AAGCC
**SRC-pGFP**	*Src -F* (HindIII)<br>GCTCA AGCTT ATGGG GAGCA GCAAG AGCAA GCCCA AGGAC<br>*Src- R* (BamHI)<br>CGGTG GATCC CCTAG GTTCT CTCCA GGCTG GTACT GGGGC
**SRCY527F-pGFP**	*Src -F* (HindIII)<br>GCTCA AGCTT ATGGG GAGCA GCAAG AGCAA GCCCA AGGAC<br>*Src-Short-R* (BamHI)<br>CGGTG GATCC CCTAG GTTCT CTCCA GGCTG
**SRCΔSH2-pGFP**	*Src -F* (HindIII)<br>GCTCA AGCTT ATGGG GAGCA GCAAG AGCAA GCCCA AGGAC<br>*Src-S1-R* (KpnI)

	CCGCG GTACC GATGG AGTCT GAGGG CGCGA CATAG TTACT<br>*Src-S2-F* (KpnI)<br>CGACG GTACC TGCCC CACGT CCAAG CCCCA GACCC AGGGA<br>*Src- R* (BamHI)<br>CGGTG GATCC CCTAG GTTCT CTCCA GGCTG GTACT GGGGC
**SRCY527FΔSH2-pGFP**	*Src -F* (HindIII)<br>GCTCA AGCTT ATGGG GAGCA GCAAG AGCAA GCCCA AGGAC<br>*Src-S1-R* (KpnI)<br>CCGCG GTACC GATGG AGTCT GAGGG CGCGA CATAG TTACT<br>*Src-S2-F* (KpnI)<br>CGACG GTACC TGCCC CACGT CCAAG CCCCA GACCC AGGGA<br>*Src-Short-R* (BamHI)<br>CGGTG GATCC CCTAG GTTCT CTCCA GGCTG

Wu_Supplemental Table 3: Based on the percentage of mobile fraction of SRCY527F-GFP and GFP-SRC(SH2) proteins within each single focal adhesion, the number of measured focal adhesions are classified into four groups and χ^2 test was conducted.

	**number of focal adhesions**	
**Mobile fraction**	SRCY527F-GFP	GFP-SRC(SH2)
**< 70%**	6	7
**70 ~ 80%**	35	11
**80 ~ 90%**	28	22
**> 90%**	9	14

$\chi^2 = 10.39$, $p = 0.0156$ ($p < 0.05$)
